# Residency Specialty and National Resident Matching Program Outcomes as Predictors of Academic vs Non-Academic Position as an Attending Physician

**DOI:** 10.7759/cureus.9548

**Published:** 2020-08-04

**Authors:** David R Hallan, Daniella Mikhail, Kimberly Lu, April Henry, Kevin Chiang, Melanie Patterson, Surav M Sakya

**Affiliations:** 1 Neurosurgery, Penn State Health Milton S. Hershey Medical Center, Hershey, USA; 2 Medicine, Penn State College of Medicine, Hershey, USA

**Keywords:** specialty, outcomes, residency, academic, non-academic, college of medicine, medical school, penn state, research

## Abstract

Purpose: Previous studies have shown that research can be used as a predictive factor for an academic career for physicians in the fields of radiation oncology, orthopedic surgery, and diagnostic radiology. We seek to determine if this factor is predictive for all medical specialties based on an analysis of public data on physicians who have trained at Hershey Medical Center (HMC) and public National Resident Matching Program (NRMP) charting outcomes.

Methods: We determined the location and job title of all graduates of HMC residency training programs through a combination of publicly available information on HMC's website and other institutions' websites. We separated these into academic and non-academic positions and performed Chi-square analysis to determine if the number of research experiences was predictive of an academic career.

Results: Participating in the residency specialties of general surgery, pathology, internal medicine, and neurological surgery are statistically significant predictors of an academic career upon graduation. The average number of research experiences obtained by matched U.S. medical students is not a statistically significant predictor of an academic career upon graduation.

Conclusion: In contrast to previously published studies, a higher number of research experiences in medical school is not a significant predictor of an academic career for attending physicians who graduated residency at HMC.

## Introduction

Academic physicians serve many roles: they are educators, investigators, and clinicians. As a result, academic physicians instruct and mentor future generations of physicians. Their research and accomplishments promote novel and improved treatments and procedures, as well as discoveries for both common and rare diseases, thereby improving the health and care of their patients. There are many factors that go into choosing between an academic versus a non-academic medical career [1,2]. A previous study of radiation oncology physicians has shown that they are twice as likely to choose an academic career if they had published at least one peer-reviewed paper during medical school [3]. Studies performed in orthopedic surgery, diagnostic radiology, and one performed that encompassed all specialties have likewise shown a positive correlation between research and an academic career [1,2,4]. Another study has found no such correlation to exist [5]. The aim of this study is to investigate if the number of research experiences that a medical student has based on the averages reported in the National Resident Matching Program (NRMP) charting outcomes of 2018 by specialty could be used to predict an academic career after completion of residency. The authors hypothesize that physicians in the specialties with the highest number of research experiences will go into academic medicine. In addition, it is hypothesized that physicians in the top five specialties with the highest number of research experiences will be more likely to go into academics, given that previous papers had shown research to be a major contributing factor to future academic positions [1-3].

## Materials and methods

Publicly available data published on Hershey Medical Center's (HMC) websites for different residency programs were used to identify past residents. Residents from the following programs were included: Anesthesiology (including Anesthesiology/Research), Dermatology, Diagnostic Radiology, Emergency Medicine, Family Medicine - Hershey, Family Medicine - University Park, General Surgery, Internal Medicine, Medicine/Pediatrics, Neurology, Neurological Surgery, Obstetrics and Gynecology, Ophthalmology, Orthopedics and Rehabilitation, Otolaryngology, Pathology, Plastic Surgery, Physical Medicine and Rehabilitation, Psychiatry, Urology, and Vascular Surgery. The residency website data included each resident's name, graduation year, and degree (MD or DO), as well as his or her employed position and geographic location. However, the position and location were often outdated, especially for residents who graduated less recently. Graduates were subsequently searched for using Google to find the most recent information on their current career and location. Institution-specific websites and physician profile websites were frequently used, as well as LinkedIn. All information was reviewed and confirmed by three separate reviewers. In this study, "academic" was defined as assistant professor, associate professor, professor, endowed chair, distinguished professor, adjunct professor, instructor, lecturer, or fellow. Non-academic roles included, but were not limited to, private practice, health groups, and the military. Information was likewise obtained from publicly available 2018 National Resident Matching Program charting outcomes on the number of research experiences that a U.S. medical graduate who matched into each medical specialty averaged. Institutional review board (IRB) exemption was granted.

Chi-square analysis was performed on the categorical variables obtained above to create 2x2 tables. P-values and Chi-square statistics were calculated without Yates correction. Significance was defined at p-value < 0.05.

## Results

Table 1 depicts the baseline data and results of HMC resident graduates by academic and non-academic positions as a function of specialty type. The specialty with the lowest percentage of those who went on to academia is emergency medicine, with 3.2% going on to academic positions and 96.8% going on to non-academic positions (p-value < 0.00001). The specialty with the highest percentage of those who went on to academia is neurological surgery, with 83% going on to academic positions and 17% going on to non-academic positions (p-value = 0.001142).

**Table 1:** Hershey Medical Center residency specialty graduates by academic vs non-academic position

Residency Program	Total	Category	
Anesthesiology		Academia - n (%)	Non-Academia - n (%)
	199	37 (19)	162 (81)
Dermatology		Academia - n (%)	Non-Academia - n (%)
	74	15 (20)	59 (80)
Diagnostic Radiology		Academia - n (%)	Non-Academia - n (%)
	14	3 (21)	11 (79)
Emergency Medicine		Academia - n (%)	Non-Academia - n (%)
	92	3 (3)	89 (97)
Family Medicine - Hershey		Academia - n (%)	Non-Academia - n (%)
	19	4 (21)	15 (79)
Family Medicine - State College		Academia - n (%)	Non-Academia - n (%)
	9	1 (11)	8 (89)
General Surgery		Academia - n (%)	Non-Academia - n (%)
	62	31 (50)	31 (50)
Internal Medicine		Academia - n (%)	Non-Academia - n (%)
	49	29 (59)	20 (41)
Medicine/Pediatrics		Academia - n (%)	Non-Academia - n (%)
	75	14 (19)	61 (81)
Neurology		Academia - n (%)	Non-Academia - n (%)
	44	17 (39)	27 (61)
Neurosurgery		Academia - n (%)	Non-Academia - n (%)
	6	5 (83)	1 (17)
Obstetrics/gynecology		Academia - n (%)	Non-Academia - n (%)
	39	11 (28)	28 (72)
Ophthalmology		Academia - n (%)	Non-Academia - n (%)
	29	8 (28)	21 (72)
Orthopedics		Academia - n (%)	Non-Academia - n (%)
	90	17 (19)	73 (81)
Otolaryngology		Academia - n (%)	Non-Academia - n (%)
	22	7 (32)	15 (68)
Pathology		Academia - n (%)	Non-Academia - n (%)
	24	12 (50)	12 (50)
Plastic Surgery		Academia - n (%)	Non-Academia - n (%)
	66	15 (23)	51 (77)
Physical Medicine and Rehabilitation		Academia - n (%)	Non-Academia - n (%)
	8	2 (25)	6 (75)
Psychiatry		Academia - n (%)	Non-Academia - n (%)
	23	9 (39)	14 (61)
Urology		Academia - n (%)	Non-Academia - n (%)
	6	2 (33)	4 (67)
Vascular Surgery		Academia - n (%)	Non-Academia - n (%)
	4	2 (50)	2 (50)
Total	954	244	710
				Percentage	100%	26%	74%

**Figure 1:**
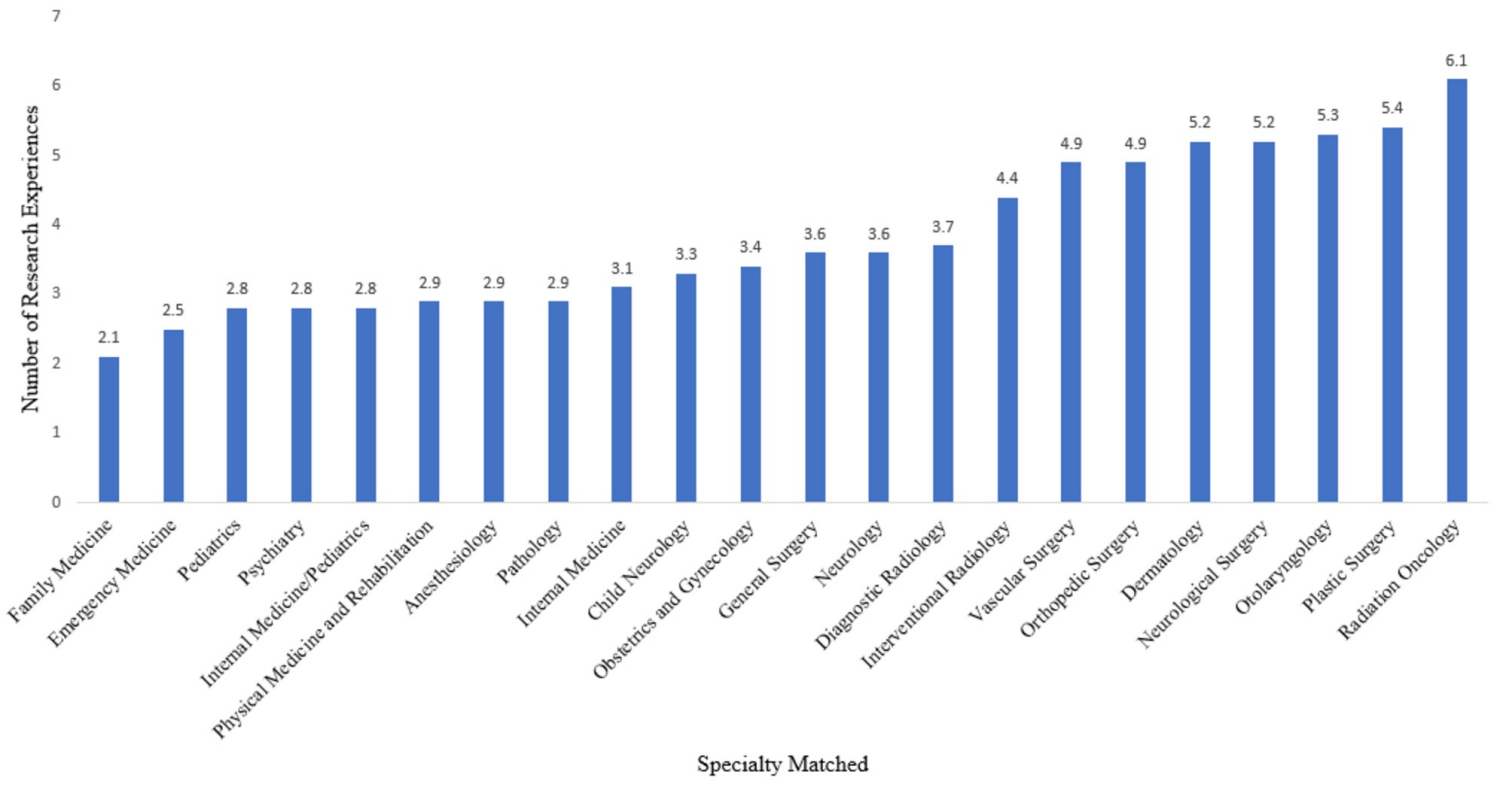
Number of research experiences as a function of residency specialty matched into by 2018 U.S. medical students

Figure 1 depicts the baseline data and results of the 2018 NRMP Match for U.S. medical students by the number of research experiences as a function of specialty choice. The top five specialties with the highest number of average research experiences are (1) radiation oncology at 6.1, (2) plastic surgery at 5.4, (3) otolaryngology at 5.3, (4) neurological surgery at 5.2, and (5) dermatology at 5.2. Because HMC does not have a radiation oncology residency program, for the purposes of analysis of the top five specialties, we replaced radiation oncology with the sixth-highest specialty, orthopedic surgery, with an average of 4.9 research experiences. Vascular surgery also had an average of 4.9 research experiences but had less data than did orthopedics.

Table 2 depicts Chi-square analyses that look at associations between specialty choice and academic vs non-academic positions obtained after residency. Statistically significant results are found in the specialties of anesthesiology (p-value = 0.011142), emergency medicine (p-value < 0.00001), general surgery (p-value < 0.00001), internal medicine (p-value < 0.00001), neurology (p-value = 0.04205), neurological surgery (p-value = 0.001142), and pathology (p-value = 0.005476). Not statistically significant results are found in the specialties of dermatology (p-value = 0.276007), diagnostic radiology (p-value = 0.720066), family medicine (p-value = 0.341967), medicine/pediatrics (p-value = 0.153031), obstetrics and gynecology (p-value = 0.700838), ophthalmology (p-value = 0.801106), orthopedic surgery (p-value = 0.126502), otolaryngology (p-value = 0.497205), plastic surgery (p-value = 0.582379), physical medicine and rehabilitation (p-value = 0.97006), psychiatry (p-value = 0.131509), urology (p-value = 0.662205), and vascular surgery (p-value = 0.261883). Notably, being in the top-five specialties for average number of research experiences was found not to be statistically significant (p-value = 0.243069).

**Table 2:** Chi-square analysis for association between residency specialty graduates and academic vs non-academic attending position observed cell totals (the expected cell totals) {the chi-square statistic for each cell}

	Top 5 Specialty in Research Experiences	Not Top 5 Specialty in Research Experiences	Row Total
Academia	59 (65.99) {0.74}	185 (178.01) {0.27}	244
Non-Academia	199 (192.01) {0.25}	511 (517.99) {0.09}	710
Column Total	258	696	954 (Grand Total)
The chi-square statistic is 1.3627. The p-value is .243069. Not significant at p < .05.			
	Anesthesiology	Not Anesthesiology	Row Total
Academia	37 (50.9) {3.79}	207 (193.1) {1}	244
Non-Academia	162 (148.1) {1.3}	548 (561.9) {0.34}	710
Total	199	755	954 (Grand Total)
The chi-square statistic is 6.4425. The p-value is .011142. Significant at p < .05.			
	Dermatology	Not Dermatology	Row Total
Academia	15 (18.93) {0.81}	229 (225.07) {0.07}	244
Non-Academia	59 (55.07) {0.28}	651 (654.93) {0.02}	710
Column Total	74	880	954 (Grand Total)
The chi-square statistic is 1.1866. The p-value is .276007. Not significant at p < .05.			
	Diagnostic Radiology	Not Diagnostic Radiology	Row Total
Academia	3 (3.58) {0.09}	241 (240.42) {0}	244
Non-Academia	11 (10.42) {0.03}	699 (699.58) {0}	710
Column Total	14	940	954 (Grand Total)
The chi-square statistic is 0.1284. The p-value is .720066. Not significant at p < .05.			
	Emergency Medicine	Not Emergency Medicine	Row Total
Academia	3 (23.53) {17.91}	241 (220.47) {1.91}	244
Non-Academia	89 (68.47) {6.16}	621 (641.53) {0.66}	710
Column Total	92	862	954 (Grand Total)
The chi-square statistic is 26.6377. The p-value is < 0.00001. Significant at p < .05.			
	Family Medicine	Not Family Medicine	Row Total
Academia	5 (7.16) {0.65}	239 (236.84) {0.02}	244
Non-Academia	23 (20.84) {0.22}	687 (689.16) {0.01}	710
Column Total	28	926	954 (Grand Total)
The chi-square statistic is 0.903. The p-value is .341967. Not significant at p < .05.			
	General Surgery	Not General Surgery	Row Total
Academia	31 (15.86) {14.46}	213 (228.14) {1.01}	244
Non-Academia	31 (46.14) {4.97}	679 (663.86) {0.35}	710
Column Total	62	892	954 (Grand Total)
The chi-square statistic is 20.7797. The p-value is < 0.00001. Significant at p < .05.			
	Internal Medicine	Not Internal Medicine	Row Total
Academia	29 (12.53) {21.64}	215 (231.47) {1.17}	244
Non-Academia	20 (36.47) {7.44}	690 (673.53) {0.4}	710
Column Total	49	905	954 (Grand Total)
The chi-square statistic is 30.6484. The p-value is < 0.00001. Significant at p < .05.			
	Medicine/Pediatrics	Not Medicine/Pediatrics	Row Total
Academia	14 (19.18) {1.4}	230 (224.82) {0.12}	244
Non-Academia	61 (55.82) {0.48}	649 (654.18) {0.04}	710
Column Total	75	879	954 (Grand Total)
The chi-square statistic is 2.0418. The p-value is .153031. Not significant at p < .05.			
	Neurology	Not Neurology	Row Total
Academia	17 (11.25) {2.93}	227 (232.75) {0.14}	244
Non-Academia	27 (32.75) {1.01}	683 (677.25) {0.05}	710
Column Total	44	910	954 (Grand Total)
The chi-square statistic is 4.1332. The p-value is .04205. Significant at p < .05.			
	Neurosurgery	Not Neurosurgery	Row Total
Academia	5 (1.53) {7.83}	239 (242.47) {0.05}	244
Non-Academia	1 (4.47) {2.69}	709 (705.53) {0.02}	710
Column Total	6	948	954 (Grand Total)
The chi-square statistic is 10.5815. The p-value is .001142. Significant at p < .05.			
	Obstetrics and Gynecology	Not Obstetrics and Gynecology	Row Total
Academia	11 (9.97) {0.11}	233 (234.03) {0}	244
Non-Academia	28 (29.03) {0.04}	682 (680.97) {0}	710
Column Total	39	915	954 (Grand Total)
The chi-square statistic is 0.1476. The p-value is .700838. Not significant at p < .05.			
	Ophthalmology	Not Ophthalmology	Row Total
Academia	8 (7.42) {0.05}	236 (236.58) {0}	244
Non-Academia	21 (21.58) {0.02}	689 (688.42) {0}	710
Column Total	29	925	954 (Grand Total)
The chi-square statistic is 0.0635. The p-value is .801106. Not significant at p < .05.			
	Orthopedic Surgery	Not Orthopedic Surgery	Row Total
Academia	17 (23.02) {1.57}	227 (220.98) {0.16}	244
Non-Academia	73 (66.98) {0.54}	637 (643.02) {0.06}	710
Column Total	90	864	954 (Grand Total)
The chi-square statistic is 2.3349. The p-value is .126502. Not significant at p < .05.			
	Otolaryngology	Not Otolaryngology	Row Total
Academia	7 (5.63) {0.34}	237 (238.37) {0.01}	244
Non-Academia	15 (16.37) {0.12}	695 (693.63) {0}	710
Column Total	22	932	954 (Grand Total)
The chi-square statistic is 0.4609. The p-value is .497205. Not significant at p < .05.			
	Pathology	Not Pathology	Row Total
Academia	12 (6.14) {5.6}	232 (237.86) {0.14}	244
Non-Academia	12 (17.86) {1.92}	698 (692.14) {0.05}	710
Column Total	24	930	954 (Grand Total)
The chi-square statistic is 7.7151. The p-value is .005476. Significant at p < .05.			
	Plastic Surgery	Not Plastic Surgery	Row Total
Academia	15 (16.88) {0.21}	229 (227.12) {0.02}	244
Non-Academia	51 (49.12) {0.07}	659 (660.88) {0.01}	710
Column Total	66	888	954 (Grand Total)
The chi-square statistic is 0.3024. The p-value is .582379. Not significant at p < .05.			
	Physical Medicine and Rehabilitation	Not Physical Medicine and Rehabilitation	Row Total
Academia	2 (2.05) {0}	242 (241.95) {0}	244
Non-Academia	6 (5.95) {0}	704 (704.05) {0}	710
Column Total	8	946	954 (Grand Total)
The chi-square statistic is 0.0014. The p-value is .97006. Not significant at p < .05.			
	Psychiatry	Not Psychiatry	Row Total
Academia	9 (5.88) {1.65}	235 (238.12) {0.04}	244
Non-Academia	14 (17.12) {0.57}	696 (692.88) {0.01}	710
Column Total	23	931	954 (Grand Total)
The chi-square statistic is 2.2746. The p-value is .131509. Not significant at p < .05.			
	Urology	Not Urology	Row Total
Academia	2 (1.53) {0.14}	242 (242.47) {0}	244
Non-Academia	4 (4.47) {0.05}	706 (705.53) {0}	710
Column Total	6	948	954 (Grand Total)
The chi-square statistic is 0.1909. The p-value is .662205. Not significant at p < .05.			
	Vascular Surgery	Not Vascular Surgery	Row Total
Academia	2 (1.02) {0.93}	242 (242.98) {0}	244
Non-Academia	2 (2.98) {0.32}	708 (707.02) {0}	710
Column Total	4	950	954 (Grand Total)
The chi-square statistic is 1.2588. The p-value is .261883. Not significant at p < .05.			

**Figure 2:**
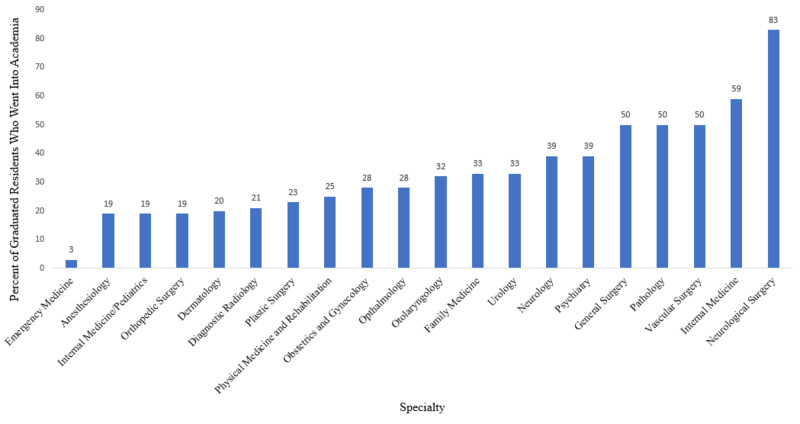
Percentage of graduated residents who went into academia as a function of specialty

The previously determined p-values are in reference to the data found in Table 1, which has been visually represented in Figure 2. Thus, for the statistically significant results found in anesthesiology, emergency medicine, general surgery, internal medicine, neurology, neurological surgery, and pathology, it can be said that 19%, 3%, 50%, 59%, 39%, 83%, and 50% of residents enter academia as attendings, respectively, which average 2.9, 2.5, 3.6, 3.1, 3.6, 5.2, and 2.9 research experiences by matched U.S. MD medical students, respectively.

## Discussion

The null hypothesis is not rejected, and average research by specialty is not a statistically significant indicator of academic career upon residency graduation. Likewise, the null hypothesis is not rejected for the top-five specialties with the most research experiences, with a p-value of 0.243069, making it not statistically significant. Instead, being in the residency specialties of general surgery, pathology, internal medicine, and neurological surgery are statistically significant predictors of an academic career upon graduation. The exact reasons of this finding are unclear, however, the authors believe that that this could be because of the two years of research required in general surgery residency at our institution, neurosurgery being a very academic and research-heavy field [6], concerns about the pathology job market being prevalent [7], and the large variety of fellowships available to Internal Medicine graduates. It could also simply be the result of a single institution's experience that, despite being statistically significant for this institution, may not generalize to a national level. These results are in contrast to the findings of Fan et al., Grimm et al., McClelland et al., and Andriole and Jeffe, who found research to be a predictive factor for an academic position upon graduation in orthopedic surgery, diagnostic radiology, radiation oncology, and graduates of all specialties, respectively [1-4]. However, it is in line with a previous study showing no association between research interest and academic careers [5].

Fan et al.'s study looked at 60 orthopedic resident graduates, exploring the factors of clerkship honors, Step 1 score, Step 2 score, number of publications prior to residency, number of publications during residency, Alpha Omega Alpha (AOA) honor society membership, additional doctorates or masters degrees, and orthopedic board examination scores in relationship to academic career. They found that the residents who went into academics had, on average, 2.36 publications compared to 0.38 publications for those who went into non-academic careers (p = 0.019) [1].

Grimm et al.'s study looked at 336 radiology resident graduates, exploring the factors of clerkship grades, Step 1 score, Step 2 score, advanced degrees, publications in undergraduate education, publications in medical school, and AOA membership. They found that the residents who went into academics had, on average, 2.7 publications during medical school compared to 1.1 for those who went into non-academic careers (p = 0.009) [2].

McClelland et al.'s study looked at 163 radiation oncology residency graduates, exploring pre-residency peer-reviewed publications and subsequent academic position versus non-academic position. They stratified their data into either having no peer-reviewed publications, one peer-reviewed publication, or more than one peer-reviewed publication. They found that those with one or more peer-reviewed publications were 3.3 times more likely to go into an academic career (p < 0.01), but those having one publication versus more than one publication did not change the likelihood of subsequent academic versus non-academic position (p = 0.7, odds ratio (OR) 1.2) [3]. 

Andriole and Jeffe's study looked at 113,522 graduates, exploring a research paper being submitted for publication during medical school (yes or no) as a predictor of future academic or non-academic career, and found that those with one publication submission in medical school were more likely to go into an academic career (p < 0.01, OR 1.3) [4].

Markert et al.'s study looked at 340 medical graduates through a survey exploring 29 different predictive factors, including "interest in research" as a medical student, resident, or fellow, as a predictor of academic vs non-academic career. They found no statistical relationship between interest in research in any training position and subsequent academic vs non-academic position [5].

In addition to research, a review of the literature shows that many factors are integral in predicting a physician's decision to pursue an academic or non-academic career. In 1988, Alpert and Coles found that competition of funding in academic positions, additional administrative demands, and demanding teaching schedules made physicians less likely to pursue an academic career [8]. Similarly, in 2002, Ley and Rosenberg found that grant funding and lower pay were significant factors, as well as debt amount and uncertainty of success [9]. Studies specifically looking at dermatologists have found that increased bureaucracy, lower pay, lack of awareness of academic positions, insufficient research resources, poor mentorship, and length of training discouraged graduating physicians from pursuing an academic career [10-13].

Limitations of this study include data collected from a single institution being compared with data collected from across the United States. Furthermore, knowing an average number of research experiences by specialty instead of having individual data, in addition to a low number of residency graduates for some specialties within HMC, is another limitation of this study. As with any observational or non-randomized study, extraneous variables can always influence an outcome, especially since a reference population is not available. These limitations could be resolved through a large-scale survey of attendings over many institutions, including both academic and non-academic, by obtaining access to the individual data that the NRMP uses to compile their annual national statistics, or through collection in a separate data registry with additional data points for matching variables that could act as confounders.

## Conclusions

The specialties of general surgery, pathology, internal medicine, and neurological surgery are statistically significant predictors of an academic career as a physician. A higher number of research experiences in medical school is not a significant predictor. Further data should be collected from other institutions in order to determine statistically significant predictors and confirm or refute this disparate conclusion, either through a large-scale survey of individuals from many academic and non-academic institutions across the United States or through data collection in registry format.
